# Enfermedades culturales de Taxco, México

**DOI:** 10.15446/rsap.V26n2.112074

**Published:** 2024-03-01

**Authors:** Adrián Urióstegui-Flores

**Affiliations:** 1 AU: Lic. Geografía. Ph. D. Geografía. Universidad Autónoma de Guerrero. Guerrero, México. a_uriostegui@yahoo.com Universidad Autónoma de Guerrero Universidad Autónoma de Guerrero Guerrero México

**Keywords:** Síndromes de filiación cultural, medicina tradicional de las Américas, terapias complementarias *(fuente: DeCS, BIREME)*, Culture-bound syndromes, traditional medicine of the Americas, complementary therapies *(source: MeSH, NLM)*

## Abstract

**Objetivo:**

Registrar A) las enfermedades culturales de Taxco de Alarcón, así como B) las causas y los tratamientos de dichas afecciones.

**Material y Métodos:**

La investigación es de tipo cualitativo no probabilístico. Se utilizó investigación documental, informantes clave, grupo focal y entrevistas a profundidad. El trabajo se realizó en la ciudad de Taxco de Alarcón, ubicada en el estado de Guerrero, en México.

**Resultados:**

Se registraron 36 enfermedades culturales.

**Conclusiones:**

Como se confirmó, la población blanco entrevistada sigue atendiendo de manera cotidiana diversas enfermedades culturales, las cuales forman parte de una válida y actual cosmovisión médica tradicional.

## Abstract (en)

**Objective:**

Record A) the cultural illnesses of Taxco de Alarcón, as well as B) the causes and treatments of said illnesses.

**Material and Methods:**

The research is qualitative, non-probabilistic. Documentary research, key informants, focus groups and in-depth interviews were used. The work was carried out in the city of Taxco de Alarcón, located in the estado de Guerrero, in México.

**Results:**

36 cultural diseases were recorded.

**Conclusions:**

As confirmed, the population interviewed continues to deal with various cultural diseases on a daily basis, which are part of a valid and current traditional medical worldview.

Existen diferentes conceptos para nombrar, categorizar o estudiar a la enfermedad o grupos de enfermedades desde el punto de vista de las ciencias de la salud, la salud pública y las ciencias sociales. Por lo tanto y en primer lugar, en el presente trabajo el concepto de enfermedad cultural lo entendemos como un término similar y alternativo a los ya definidos por los descriptores de las ciencias de la salud (DECS/MESH) 1, y el cual se explica a continuación: "En medicina y antropología médica, un síndrome ligado a la cultura, un síndrome específico de una cultura, síndrome cultural o una enfermedad popular es una combinación de síntomas psiquiátricos y somáticos que se consideran una enfermedad reconocible solo dentro de una sociedad o cultura específica. No hay alteraciones bioquímicas o estructurales objetivas de los órganos o funciones del cuerpo, y la enfermedad no se reconoce en otras culturas" 1.

Sin embargo, del concepto citado en el párrafo anterior discuto donde se menciona que "la enfermedad no se reconoce en otras culturas" 1; aquí solo agregamos que, a nuestro parecer, algunas enfermedades culturales pueden tener ciertas similitudes o variantes semejantes en diferentes sociedades, grupos y categorías sociales, e incluso puede haber un proceso de difusión, de reinterpretación y de modificación (conceptual, terapéutica e ideológica) de dichas afecciones por parte de culturas, subculturas o estratos sociales que han sido receptores de dicha difusión cultural.

En el mismo sentido, en escritos recientes 1 es posible encontrar diversos términos alternativos de enfermedades culturales, tales como "enfermedades específicas de la cultura, enfermedades específicas de una cultura, enfermedades ligadas a la cultura, enfermedades tradicionales, enfermedades de filiación cultural, síndromes culturales, síndromes específicos de la cultura, síndromes específicos de una cultura, síndromes ligadas a la cultura, síndromes relacionadas a la cultura, trastornos culturales, trastornos específicos de la cultura [o bien] trastornos específicos de una cultura" 1.

También se encuentran definiciones tales como entidad nosológica, nosotaxia sociocultural (2 p. 125), nosología de móvil mercantil, disease mongering (2 p. 129); o las ya citadas por Zolla et al. (3 p. 13-32) tales como enfermedades o nosologías de la medicina tradicional, cultura médica de las clases subalternas, expresiones médicas populares o tradicionales, nosotaxia popular, enfermedades del curandero, respuestas sociales a la enfermedad (en los ámbitos de la medicina doméstica, de la medicina tradicional y de la medicina académica), enfermedades tradicionales, enfermedades folk, supersticiones, enfermedades de los terapeutas tradicionales, epidemiología de la medicina tradicional, enfermedades naturales, enfermedades personales y enfermedades sobrenaturales, enfermedades de Dios, narangic, enfermedades por frío o calor, enfermedades naturales y enfermedades preternaturales, o bien enfermedades por sus manifestaciones patológicas, por la causa o por el ámbito de la demanda (3 p. 13-32).

Asimismo, se mencionan conceptos tales como nosología popular, padecimientos, síndromes cultural-mente delimitados, enfermedades tradicionales (4 p. 83); enfermedades naturales, sobrenaturales, de los dioses del cielo, de la tierra, de los dioses del mundo inferior, de los dioses de linaje y ancestrales, hechicería, nagualismo, cortar la hora (5 p. 118-154); o bien la distinción entre enfermedad (disease) y padecimiento (illness) (6 p. 47).

Dentro de la misma temática, se aluden términos tales como "síndromes culturales latinoamericanos" y su explicación detallada (7, p. 329-348); entidades nosológicas tradicionales o populares (8, p. 400); medicina invisible, perfil epidemiológico invisible (8, p. 401), o bien las principales nosologías de la medicina tradicional (9, p. 210-211).

Como se confirmó, ya se ha explicado el concepto de enfermedad entendida desde la cosmovisión de diversos grupos étnicos de México (10 p. 1), y la cual puede ser provocada por diferentes tipos de sucesos, tales como la pérdida de alma, la pérdida de sangre debido a accidentes, una fractura, la intrusión mágica de objetos, la penetración de algún aire, la intrusión de una esencia proveniente de otra persona, debido al coraje, o por la penetración en el organismo de sustancias frías o calientes (10 p. 1), por citar solo algunos ejemplos.

Además, se encuentra la noción de centros anímicos (11, p. 197-220); entidades anímicas (11, p. 221-262); lo frío y lo caliente (12, p. 16-22); o bien, la doctrina del humorismo (12, p. 22-26).

A nuestro parecer, las enfermedades culturales también pueden entenderse como creencias, mitos, ideas populares, e inclusive como una traducción o interpretación popular de las enfermedades biomédicas (o de sus signos y síntomas derivados).

De manera similar, consideramos que las enfermedades culturales pueden ser analizadas desde el panorama del modelo médico hegemónico, el modelo alternativo subordinado y el modelo de autoatención (13, p. 97-111); a partir de los sistemas médicos profesionales tradicionales tales como la medicina aryuvédica, la medicina China, la medicina galénica, la medicina unani, la medicina tradicional no profesional, o la biomedicina (14, p. 15-21); desde el modelo clásico, el modelo pragmático o el modelo crítico (15, p. 11-44); o bien "a partir de orientaciones interpretativas, hermenéuticas, semióticas, narrativas e incluso estéticas" (15, p. 82).

Como se ratificó, en trabajos especializados 16 ya se han analizado diversos "síndromes de filiación cultural" en dicha ciudad de Taxco, tales como mal de ojo, empacho, aire, caída de mollera, susto, envidia, embrujo, sangre con toxinas, etika, algodoncillo, púrpura, niños enlechados y aquiztle.

Finalmente, existe una gran cantidad de obras científicas que han estudiado, analizado o citado varias enfermedades culturales, ya sea por grupos de afecciones o de manera individual 17-26,28-31.

## MATERIAL Y METODOS

**Figura 1:**
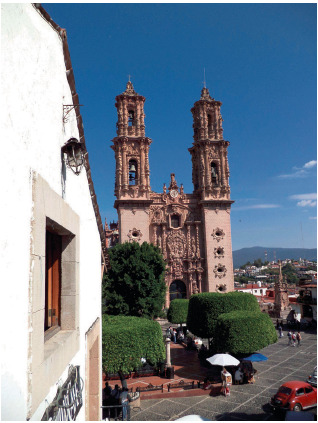
La iglesia de Santa Prisca. Taxco de Alarcón, México

El trabajo se realizó en la ciudad de Taxco de Alarcón, ubicada en el estado de Guerrero, en México. Para el año 2020 dicha localidad contaba con 50 399 habitantes 27 (Figuras 1 y 2).

**Figura 2:**
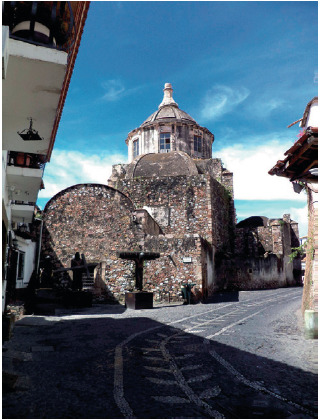
Exconvento de San Bernardino. Taxco de Alarcón, México

Se utilizó investigación documental, informantes clave, grupo focal y entrevistas a profundidad. Se diseñó un cuestionario de cinco preguntas y se entrevistó a cuatro médicos tradicionales, y a un grupo focal compuesto por 12 personas. En total, se entrevistó a 16 habitantes. La investigación es de tipo cualitativo no probabilístico.

## RESULTADOS

Se registraron 36 enfermedades culturales. Los nombres de dichas afecciones, así como las causas y los tratamientos pueden observarse con mayor detalle en la Tabla 1.

**Tabla 1:** Enfermedades culturales registradas en Taxco de Alarcón, México

Nombre(s)	Causas	Tratamientos
Abultamientos y deformaciones en la piel y músculos provocados por envidia	Por envidia. Las personas envidiosas tienen la capacidad de dañar. Son provocadas por la envidia que le tienen a la persona, sobre todo si las personas que tienen la envidia son gemelos o mellizos.	Masajes con tintura de manzanilla o sábila en el área dañada. Masajes o “sobaduras” en la zona afectada. Rezos de la religión católica.
Aire Aire en cuerpo Aire en los músculos o en la piel Aire en ojos Aire en oídos Aire en cuello	Por pasar por donde existen almacenamientos de agua en caminos, terracerías, barrancas o donde existen aguas estancadas. Salir en la noche cuando hace frío, o después de haber comido. No cubrirse cuando hace frío, o por lluvia o viento helado. Exponerse al aire frío. Por los cambios extremos de temperatura. No cubrirse después de bañarse.	Se hace una “limpia” con las hierbas jarilla, ruda jazmín, chon de China, albahaca, santamaría, pirul y limón. Se toma té de ruda, y se coloca jarilla en el estómago con alcohol y una venda. Se utiliza manzanilla, rosa de castilla o humo de cigarros, o bien altamisa, albahaca, romero y jarilla.
Anginas	El término de anginas es el nombre popular, y en la medicina moderna se denominan “amígdalas” (10, p. 211). Exponerse a climas fríos o calurosos extremos, o bien a cambios bruscos de temperatura. Comer alimentos muy fríos o calientes. Exponerse al sol mucho tiempo.	Evitar exponerse a climas fríos o calurosos extremos, o al sol intenso. No comer alimentos muy fríos o calientes. No exponerse a cambios bruscos de temperatura.
Aumento de sangre Aumentar la sangre Mejorar la sangre	Desnutrición. Mala alimentación. Depresión. Susto. Espanto.	Hervir dos tallos con cinco a siete hojas de muicle (*Jacobinia spicigera*) por 20 minutos, y tomar como agua de tiempo durante tres a cinco días. Comer betabel (*Beta vulgaris*) en la comida, o como jugo. Comer de manera equilibrada en las horas establecidas y no “malpasarse” en los horarios de las comidas. Evitar la comida chatarra. Evitar las depresiones y estados emocionales dañinos.
Bilis	Se refiere a que se “riega” la bilis que produce el hígado, y que es almacenada en la vesícula biliar. Por enojo, disgusto o una fuerte impresión emocional. Por no comer los alimentos en la cantidad y calidad establecida, y en la hora indicada.	Comer los alimentos en la cantidad y calidad establecida, y en la hora indicada. Comer los alimentos que se desean o quieren.
Brujería	El brujo (a) puede provocar diversas enfermedades por medio de brujería, hechizos o magia dañina, y para su curación se tiene que acudir con este tipo de personas. La brujería se considera un proceso mágico y místico, e incluso se vincula con la religión. Rencor. Odio. Envidia. Mala voluntad. Venganza.	Acudir con brujos (as) para el tratamiento. Cargar una bolsa roja con ajo, un moño rojo, o un ajo macho. Cruzarse las venas (colocar las venas de los brazos en forma de cruz para que la enfermedad no llegue a la persona).
Caída de mollera	Deshidratación del niño. Por golpes o caídas del niño. Por susto o espanto del niño.	Se mete el dedo en la boca y en el paladar del niño, y se da un masaje para acomodar la mollera. Se coloca la cabeza del niño en agua para que se suba o se acomode la mollera.
Caída de ovarios Ovarios caídos	Los periodos de la mujer están desubicados. El organismo de la mujer se encuentra desubicado. Depresión. Preocupaciones graves. Emociones dañinas.	Evitar estados emocionales dañinos. No usar faja. No usar zapatos de tacón.
Caída de ovarios Ovarios caídos	Los periodos de la mujer están desubicados. El organismo de la mujer se encuentra desubicado. Depresión. Preocupaciones graves. Emociones dañinas.	Evitar estados emocionales dañinos. No usar faja. No usar zapatos de tacón.
Chamanismo (para la curación de enfermedades culturales tales como espanto o susto) Chamanismo (para la curación de depresión o de malestares emocionales)	Impresiones emocionales muy fuertes. Depresión y estados emocionales negativos causados por problemas personales, familiares o del trabajo. Estados emocionales negativos por la grave delincuencia e inseguridad que se vive en la ciudad.	Ayahuasca. Se emplea una rana venenosa y tóxica, la cuál tiene propiedades alucinógenas.
Chaneques (enfermedades culturales causadas por chaneques)	Se tiene la creencia de que “los chaneques son niños de seis años con caras de señor cuyo objetivo principal es el juego y pueden hacer daño en día número non con moretones, golpes o bien seguir a la persona. Se encuentran principalmente en piletas, ríos, pozos de agua, huertos o donde están las hojas grandes” (31, p. 77). Los chaneques pueden provocar enfermedades.	“El agua bendita y el rezo acompañado de un borracho o una prostituta en el lugar donde han sido observados son las principales medidas de protección” (31, p. 77).
Ciclos lunares que afectan el parto Daño en mujeres embarazadas y en el bebé por exponerse a diversos ciclos lunares o a la luna llena Malestares por luna llena	El exponerse a ciclos lunares o la luna llena puede provocar enfermedades físicas y emocionales en mujeres embarazadas y en el bebé. Se tiene la creencia de que la luna llena tiene una energía dañina que puede afectar a mujeres embarazadas y a bebés en el periodo del embarazo. La exposición a la luna llena también puede provocar espanto, susto, o emociones dañinas graves que provocan enfermedades físicas y emocionales. Se considera que el clima frío, la lluvia o el viento helado en los ciclos lunares o en luna llena puede provocar enfermedad en la mujer embarazada y en el bebé en gestación.	No exponerse por mucho tiempo a los ciclos lunares o a la luna llena. Cubrirse del frío, lluvia o viento cuando hay ciclos lunares o luna llena.
Comer la hiel de pollo Hiel para la diabetes	La hiel de pollo sirve para curar enfermedades tales como la diabetes	Se come la hiel de pollo. Dicha hiel es una traducción de la bilis que secretan las glándulas endocrinas de dichas aves. La hiel es una traducción de la bilis que produce el hígado, y que es almacenada en la vesícula biliar.
Empacho	Alimentos que se quedan pegados, no se digieren bien, están descompuestos o no le caen bien a la persona. Comer alimentos muy secos, en demasiada cantidad o en mal estado. Sobre todo, se presenta el malestar en niños.	Se da un masaje en toda la espalda, se aprieta la piel con la yema de los dedos y se da un jalón firme y rápido; con lo anterior "se quiebra" el empacho. Se soba o se da un masaje en el estómago jalando la piel. Se soba el estómago con aceite, hierbabuena y carbonato. Tomar una cucharada de aceite de oliva. Se toma un té de manzanilla, hierbabuena y albahaca. Se acude con médicos tradicionales o con alguna persona de la ciudad que sepa curar el empacho.
Enfermedades de penitentes Daño por penitencias Daños o malestares de encruzados, flagelantes y ánimas	Son enfermedades físicas, daños o lesiones provocados específicamente por el tipo de penitencia católica que se practica en la Semana Santa en Taxco. La enfermedad o lesión depende del tipo de penitencia realizada, ya sea como encruzados, flagelantes o ánimas. Por ejemplo, del penitente denominado "encruzado" las lesiones o daños son heridas, cortadas, abrasiones y heridas punzocortantes en la espalda, cuello, brazos, manos y piel, así como daños osteomusculares; todo lo anterior causado por cargar espinas de zarza muy pesadas en la espalda, así como también heridas, cortaduras y abrasiones por caminar descalzos. De los "flagelantes" son heridas e infecciones en la espalda por golpearse con lazos y clavos de hierro; lesiones osteomusculares y de la piel por cargar cruces de madera pesadas; y heridas, cortaduras y abrasiones por caminar descalzos por las calles durante todas las procesiones. De las "ánimas" (que son penitencias que realizan las mujeres) son lesiones osteomusculares en la espalda, cadera, rodillas, tobillos y pies, sobre todo por caminar agachadas arrastrando cadenas en los pies y cargando cruces de madera pesadas. Y también heridas, cortadas, abrasiones y lesiones en los pies.	Consulta con médicos generales o especialistas. Se aplican antibacteriales y antivirales por medio de autoatención, ya sea por experiencia propia de familiares o amigos que han realizado las penitencias, o de consultas previas tenidas por dichos penitentes con médicos generales o especialistas. Se aplica alcohol o yodo en las lesiones. Consulta con médicos generales o especialistas. Se aplican tratamientos con hierbas medicinales, tanto untados (pomadas, tinturas o cremas) o tomados en té. Consulta con médicos generales o especialistas.
Enfermedades por desobedecer normas, compromisos, promesas o rituales de la secta de la Santa Muerte	Desobedecer las normas, compromisos, promesas o rituales de dicha secta.	Cumplir con las normas, compromisos, promesas y rituales. Acudir con una de las personas que dirige la secta en el Barrio de Minas Viejas, ubicado en la periferia de la ciudad. Dicho dirigente emplea rituales, oraciones, rezos y tratamientos específicos.
Enfermedades, castigos o consecuencias por no cumplir las promesas o compromisos hechos con la religión, con el santo patrono o al Cristo específico (en la ciudad cada iglesia tiene su Cristo con un nombre específico) Lo anterior se refiere específicamente a la religión católica	Se tiene la creencia de que diversos castigos o enfermedades físicas, mentales o emocionales, accidentes o heridas pueden ser causados por no cumplir las promesas, compromisos o juramentos que la persona hizo a la religión católica o a su santo patrono.	Cumplir con las promesas, compromisos o juramentos realizados. Acudir con sacerdotes católicos. Hacer y cumplir los rituales, oraciones, normas y penitencias de la religión católica.
Enfriamiento de cuerpo Enfriamiento Se enfría el cuerpo	Por salir cuando hace clima frío, viento frío o en temporada de lluvias sin cuidarse o abrigarse. Descuidarse en temporada de frío y de lluvias cuando la persona tiene diversas enfermedades. Pasar por donde hay fuentes de agua tales como barrancas, aguas estancadas o tanques grandes.	Se coloca alcohol en una cazuela de barro con un periódico encima y se pasan los pies sobre el calor que genera dicho alcohol. Posteriormente, se pone tomate asado en la planta de los pies. Rezos de la religión católica. Combinaciones de hierbas medicinales. Consulta a médicos generales y especialistas.
Envidia Daño por envidia Daño por mala voluntad Daño por odio	Si una persona tiene envidia puede provocar enfermedades. La gente que tiene envidia, odio o mala voluntad puede colocar cosas dañinas o tóxicas en los alimentos, bebidas o cigarros, las cuales pueden dañar o destruir permanentemente el cerebro, las facultades mentales u otras partes del organismo. Son colocadas sustancias químicas destructivas en las bebidas o comidas, con engaños y sin el consentimiento de la persona.	Evitar beber, comer o fumar con personas desconocidas. Acudir con médicos generales o especialistas para saber por estudios químicos la sustancia que se consumió. Acudir con médicos tradicionales.
Granos en la lengua Erupciones en la lengua o boca	Por no comer lo que se le antoja a la persona le pueden salir granos o erupciones en la lengua o en la boca.	Comer los alimentos que se le antojan a la persona o al niño.
Latido regado Pulso regado Juntar el latido Juntar el pulso	Se refiere a los desequilibrios en el sistema cardiovascular, la presión baja o alta, o desequilibrios en la presión arterial. También se origina por depresión, por hacer ejercicio extremo sin descansar, por sobreentrenamiento físico extremo, o por comer alimentos que no le caen bien a la persona.	Colocar un pedazo de migajón de pan o bolillo adentro del ombligo de una a tres noches. Y se amarra con un pedazo de tela para que no se caiga. Se tiene que juntar el pulso en el estómago o en el ombligo por medio de masajes. Debe sentirse la palpitación en el ombligo, si no se siente dicha palpitación tienen que "sobar" el estómago. Se debe "jalar" el latido con masajes. Sobaduras. El pulso se tiene que sentir en el ombligo después del masaje. Descanso del ejercicio. Acudir con médicos tradicionales que componen el pulso, o bien con especialistas tradicionales empíricos llamados "pulseros" o "juntadores del pulso o latido".
Llaga enconada	Se refiera a las heridas que se infectan por bacterias, hongos o parásitos.	Se recurre a médicos generales. Herbolaria medicinal.
Mal de ojo	Porque el niño está gracioso, "chulean" mucho a los niños, o por la envidia que se tiene al niño. Se tiene la vista fuerte sin saberlo, lo que afecta principalmente a niños y plantas. Vista muy pesada y mal intencionada que afecta a niños. Por influencias negativas de algunas personas. Por espanto, susto, aire o envidia (enfermedades culturales). Creencias. Superstición. Porque no los toca la gente que causó el malestar.	Limpiar con un huevo. Hablar en el cerebro del niño. Decir el nombre del niño tres veces cerca de su cerebro. Lamer la cabeza del bebé. Quebrar el cuerpo poniendo sal en la lengua y escupir en la frente y en el estómago, hacer una cruz con el dedo, dar apretones por todo el tronco y con ropa interior sucia limpiar la frente y todo el cuerpo. Pasar un chile guajillo o ancho por la frente y cuerpo. Emplear jarilla, ruda, hinojo y santamaría. Alumbre, un chile verde y limón. Usar un huevo y/o un fruto llamado "ojo de venado", o una bolsa de tela roja con ajo, chile ancho y coral de mar. Colocar alguna cosa de oro adentro de la ropa (en donde se descarga la vista fuerte y protege al niño). Limpiar con una camisa sucia. Jarilla, ruda, hinojo y santamaría. Pasar por el cuerpo un huevo y después quebrarlo en medio vaso de agua para ver el daño. Escupir con sal. Hacer una limpia antes de las doce del día tres veces por semana con jarilla, flor de santamaría, huevo, chile, limón y alcohol.
Mal de orín Mal de orines	El mal de orín se refiere a que la persona orina en exceso, de manera exagerada, o en una gran cantidad fuera de lo normal. Es provocada por el mal funcionamiento de partes u órganos relacionados con el sistema urinario o por diabetes. También se tiene la creencia de que es provocado por la envidia de otra persona o personas, lo que afecta una parte del organismo de la gente que es envidiada, o bien por un castigo de la religión que profesa la persona por cometer alguna falta grave. Por tomar algún veneno o sustancia tóxica que fue administrada por envidia por alguna persona, brujo o hechicero. Dicha sustancia o bebida es administrada sin el conocimiento de la persona y con engaños, o cuando se toman bebidas alcohólicas adulteradas con sustancias dañinas (con personas o grupos desconocidos). Por tomar bebidas nocivas tales como refrescos, jugos muy azucarados, exceso de alcohol o cerveza, bebidas alcohólicas preparadas, o bebidas tóxicas o contaminadas con algún compuesto nocivo. Por descuido en las comidas, las cuales no se hacen a las horas correctas, por "malpasarse" al no comer en los horarios establecidos o por no comer en la cantidad y calidad recomendadas por las personas mayores.	Se hierven dos o tres hojas y una parte del tallo de Palo de Brasil *(Haematoxylum brasiletto),* y se toman en té como agua de tiempo mientras se tenga la enfermedad. Acudir con médicos tradicionales. Rezos de la religión católica. Consultar a médicos generales y especialistas para que se hagan los análisis químicos correspondientes.
Mala voluntad	Se refiere a la mala voluntad, envidia, rencor, enojo u odio que se le tiene a la persona, y que puede provocar diversas enfermedades.	Se coloca una bolsa pequeña cerca del corazón de la persona. Dicha bolsa contiene las hierbas de pimienta y clavo, una cruz católica y la oración de san Benito. La persona debe llevar dicha bolsa en todo momento. En el caso de las mujeres, se coloca esta bolsa en la ropa interior superior. La bolsa descrita con anterioridad otorga protección de la mala voluntad, las envidias y protección para realizar el trabajo cotidiano. Acudir con médicos tradicionales, o con personal biomédico (enfermeras o médicos generales).
Modorra Desguanzado Desgano Decaimiento Ánimo caído	Esta afección se refiere a la falta de motivación, o falta de ganas para hacer las cosas cotidianas. Es causada por no tener ánimo, no tener fuerza emocional, tristeza, depresión, angustia, cansancio extremo, estrés crónico o flojera, por ejemplo. También es provocada porque la persona tiene enfermedades infecciosas o crónico-degenerativas, o la presión arterial muy baja o muy alta.	Evitar enfermedades emocionales, conflictos, enojos y depresión. Buscar el apoyo familiar. Consultar a médicos generales y especialistas. Consultar a psiquiatras. Acudir con psicólogos.
Nagual (enfermedades culturales causadas por nagual)	"Se refiere a gente que puede adquirir forma de animales locales (leopardo, venado o aves, por ejemplo)" (31, p. 77-78).	"Para conseguir protección es necesario enterrar un clavo en medio de la casa sin que nadie se entere, rezar en la vivienda, colocar ajos en forma de cruz o bien, usar flores rojas, albahaca, agua bendita, un huevo y alcohol" (31, p. 77-78).
Niños empiojados Niños con piojos	Un informante comentó que anteriormente los niños se bañaban una vez cada semana, o una vez cada 15 días. Falta de aseo. Piojos parásitos.	Se muelen dos hojas de flores de belladona *(Datura stramonium)* y se amarran con una tela sucia en el cabello del niño durante una o varias noches. Se emplean tres hojas con un tallo de belladona, se hierve con agua y se toma antes de dormir.
Niños mudos Niños que no pueden hablar	Envidia. Mal de ojo. Problemas con el aparato bucal del niño.	Se coloca un pan con chocolate en la boca del niño, sin morder. Se recomienda que el pan sea el que se dio a otros niños en su primera comunión. Se recomienda "guardar la primera sopa del niño": esto quiere decir que se guarda el pan con chocolate del niño que hizo su primera comunión en la religión católica. Se tiene que ir a una iglesia católica, y se tiene que colocar la llave del sagrario en la boca del niño (el sagrario es el lugar donde se guardan las ostias).
Pegar al bebé Componer el bebé	Es una afección de las mujeres embarazadas. La causa es que el bebé viene de cabeza o está mal ubicado.	Se calientan dos tortillas en el comal lo más que se pueda, y se coloca en el estómago de la mujer embarazada durante una noche. Sobaduras. Acudir con parteras empíricas, o con médicos generales y/o especialistas.
Postemillas	Son erupciones o úlceras en la piel o en los labios provocados por infecciones de bacterias, virus, hongos o alergias. Por estrés. Por alimentos que hacen daño.	Se utilizan hierbas curativas o medicamentos de farmacia.
Reumas	Es el dolor leve, moderado o fuerte o los problemas de movimiento en diversas partes del sistema musculoesquelético, o daños en el sistema nervioso o en la piel. Es provocado por la humedad, el clima frío, viento frío, lluvias, o por los cambios bruscos de temperatura. Por golpes, torceduras, desgarros o lesiones.	Se utiliza la marihuana *(Cannabis sativa).* Se aplica en fomentos o diluida con agua hervida para los dolores musculoesqueléticos y de las articulaciones. También algunos de los informantes la fuman para la misma finalidad. Se utiliza la manzanilla *(Helenium quadridentatum).*
Reventar la hiel Romper la hiel	Se revienta la hiel o se dañan los órganos que la producen o por donde pasa dicho compuesto químico, tales como el hígado, la vesícula biliar o el páncreas. Aquí la hiel es una traducción de la bilis que produce el hígado, y que es almacenada en la vesícula biliar. Por no comer los alimentos que se le antojan a la persona o al niño. Por ver a otra persona comer y no poder probar el alimento que está comiendo dicha persona.	Comer los alimentos que se desean o quieren. Alimentarse de manera adecuada y saludable.
Sereno Dar el sereno El sereno	Es una traducción popular sobre los factores climáticos extremos que se suscitan en la noche, tales como el clima frío, el viento helado, la humedad, las heladas o la lluvia, y los cuales pueden provocar diversas afecciones. Quedarse por mucho tiempo al aire libre en la noche. Por dormir al aire libre o a la intemperie por la noche.	No exponerse al clima frío, viento frío, humedad, lluvia o heladas en la noche. Evitar quedarse por mucho tiempo al aire libre en la noche. No dormir al aire libre o a la intemperie por la noche. Acudir con médicos generales o especialistas.
Subida del muerto Se sube el muerto Se "sube el muerto al dormir"	Se tiene la creencia de que una persona fallecida se puede subir al cuerpo de una persona que se encuentra dormida. Aquí se tiene la sensación de que la persona no se puede despertar, pero se encuentra consiente e inmóvil sin poder moverse. Es causada porque la persona tuvo un espanto o susto. Es un trastorno normal del sueño, el cual ya ha sido explicado de manera científica 30.	Administración de tratamientos de autoatención o con herbolaria medicinal. Consulta a médicos tradicionales. Consulta con médicos generales o con especialistas. Se emplea la hierba azahar *(Citrus medica).*
Susto o espanto provocado específicamente por fenómenos de la naturaleza (tales como sismos, terremotos, deslaves de cerros, fallas geológicas y/o incendios forestales)	Esta es una variante de la enfermedad cultural denominada "susto" o "espanto". La característica principal aquí es que es causada cuando hay sismos, terremotos, deslaves de cerros, fallas geológicas o incendios forestales.	Tomar té de ajenjo. Se practican rezos católicos y la persona se pone de rodillas. Acudir con sacerdotes católicos. Tratamientos con fármacos de la biomedicina. Acudir con el personal biomédico de los centros de salud locales, o con psicólogos o psiquiatras.
Susto Espanto	Por tener una fuerte impresión emocional. Problemas emocionales. Golpes. Caídas.	Té de ajenjo, manzanilla o tila. Comer un pan duro. Rezos católicos por parte de la persona, o acudir con un sacerdote católico. Acudir con médicos tradicionales, parteras empíricas, o con personas que sepan curar el espanto o susto.

Fuente: trabajo de campo.

## DISCUSIÓN

Se recopilaron 36 enfermedades culturales en Taxco, así como sus causas y tratamientos (Tabla 1); el registro anterior es la contribución científica del presente escrito a dicho tema especializado. Como se confirmó, en diversos trabajos ya se han analizado o estudiado algunas de las enfermedades culturales que se reconocieron en Taxco 1-26,28-31.

En los escritos de Villalva y Barrera 26, por ejemplo, se han examinado 10 enfermedades de filiación cultural, mientras que en los escritos de Mateos (9, p. 210-211) se abordan 10 de las principales nosologías de la medicina tradicional de México. Inclusive autores como Peretti (28, p. 18) también han definido el concepto de enfermedades culturales.

El panorama sobre las concepciones de los ciclos lunares que afectan el parto ya ha sido aludido en escritos que realizan evaluaciones formativas al Programa de Parteras Empíricas implementado por personal de la Secretaría de Salud en el municipio de Taxco (17, p. 6-9); y también otras enfermedades culturales se han observado en grupos étnicos tarascos (29, p. 87-91) tales como el empacho, la chorrera, el chorro con sangre, las afecciones de los que comen mucho, o bien por chicle o tortilla pegados en el estómago, daño por tristeza, malestares por desidiosos, o la "enfermedad por desaseo", por ejemplo.

La afección conocida como "subida del muerto", o "se te sube el muerto" ya ha sido examinada de manera científica: "Cuando una persona afirma que se le sube el muerto, se trata de un trastorno del sueño conocido como parálisis del sueño, que es común entre la población" (30, p. 1).

En el mismo sentido, las 10 principales causas de demanda de atención de la medicina tradicional (tales como el mal de ojo, empacho, susto-espanto, caída de mollera o disentería, por citar algunos ejemplos) han sido aludidas por Zolla et al. (3, p. 12). Asimismo, se ha explicado a profundidad la causa y el tratamiento de la caída de mollera en grupos étnicos nahuas del altiplano central (11, p. 250),

El "latido" es citado por Mateos (9, p. 211) y por Piedra-santa (4, p. 88); asimismo, el susto, el mal de ojo y el mal aire se han reconocido en la región Mixe Baja del estado de Oaxaca 4. El mal de orina, la modorra-cansancio, las enfermedades provocadas por brujería, enfermar de antojo, envidia, espanto, mal aire, mal de ojo y sereno ya se han catalogado en diversos grupos étnicos de México (19, p. 86-90).

Por otra parte, las ideas sobre el susto recopiladas en Taxco difieren de lo citado por Sepúlveda para grupos étnicos purépechas, ya que en dichos grupos el susto provoca la pérdida del alma, y esto puede sobrevenir de cualquier choque emocional", y también se menciona a la caída de mollera, la bilis, el mal de ojo y el empacho (19, p. 71-72).

Se presentan ciertas similitudes con la denominada "llaga enconada" observada en los purépechas (19, p. 80), ya que en Taxco la llaga enconada remite a las heridas infectadas. Asimismo, existen diferencias con la denominada "bilis" registrada en grupos purépechas, ya que remite a una "enfermedad que se presenta cuando esta sustancia que invade todo el cuerpo se coagula" (19, p. 72). En la ciudad de estudio la "bilis" se refiere a que se riega dicha bilis que produce el hígado, y que es almacenada en la vesícula biliar.

Las personas entrevistadas no mencionaron enfermedades provocadas por cualidades como frío o calor; las cuales ya han sido mencionadas por López (12, p. 16-22) en nahuas y mestizos de Morelos, Veracruz y Distrito Federal, en tarascos de Michoacán, en mayas de Yucatán, Chiapas y Quintana Roo, en otomíes del estado de México y en población de la Región de La Laguna (12, p. 16-17).

Tampoco se registró el concepto de tonalli, el cual "era una fuerza que determinaba el grado del valor anímico del individuo" (11, p. 233), ni semejanza alguna con las nociones africanas de "sombra", que "abandona el cuerpo durante el sueño y cuando la mente vagamundea" (22, p. 108-110).

Las creencias sobre "chaneques" han sido observadas desde el México prehispánico (9, p. 192-194). Lo anterior parece tener cierta semejanza con los denominados "chanes" de las tribus chichimecas del norte de México, que "castigan a quienes provocan su enojo" (22, p. 104-105). En Taxco las creencias sobre chaneques derivan de migrantes originarios de la Región Costa Grande del estado de Guerrero (Coyuca de Benítez, sobre todo), y que hoy viven en la ciudad (31, p. 77).

La noción de nagual también tiene un origen prehis-pánico y remite a la capacidad que tiene una persona para transformarse en animal, sobre todo durante la noche (9, p. 194-196). La definición anterior presenta semejanzas con lo registrado en Taxco, y se refiere a que el nagual es un habitante que puede adquirir forma de animales locales (leopardo, venado o aves, por ejemplo) (31, p. 77-78).

En el mismo sentido, López (11, p. 302-303) menciona la práctica de la confesión ante el sacerdote de Tlazoltéotl en grupos étnicos de origen náhuatl, la cual se encontraba vigente en el año de publicación de dicho escrito: "Todavía se acostumbra en poblaciones de origen náhuatl recurrir a la confesión en casos de graves enfermedades" (11, p. 302-303). A diferencia de lo anterior, varios de los informantes entrevistados manifestaron recurrir a la confesión, y a rezos y oraciones de la religión católica o protestante cristiana para buscar la curación de enfermedades culturales.

Asimismo, los sinónimos de mal de ojo tales como "mal viento", "aire malo", "ojeadura" u "ojeada", aludidos por Zolla et al. (3 p. 59), no se mencionaron en Taxco.

Finalmente, las consideraciones sobre brujería ya han sido estudiadas en grupos mesoamericanos, aztecas, mayas, huaxtecos, mixtecos, zapotecos, matlazincas, totonacos y purépechas (19, p. 64-69); por Holland (5, p. 132-154) en los Altos de Chiapas, o por Sepúlveda 18. Y autores como Aguirre (22, p. 110-114) también atribuyen a las representaciones de brujería una procedencia europea.

Para terminar, me permito discutir otro dato observado en el desarrollo del presente trabajo: en los conceptos de síndromes de filiación cultural, así como de enfermedades específicas de la cultura y de sus diversos sinónimos explicados en el DECS/MESH 1 se menciona que: "No hay alteraciones bioquímicas o estructurales objetivas de los órganos o funciones del cuerpo" 1. Sin embargo en la ciudad de Taxco registramos los términos de "enfermedades de penitentes", "daño por penitencias" y "daños o malestares de encruzados, flagelantes y ánimas", en las cuáles sí se ratificaron y confirmaron alteraciones estructurales físicas del organismo humano, e inclusive afectaron algunos de sus órganos o funciones. Todo parece indicar (a manera de hipótesis) que pueden existir enfermedades culturales (cimentadas en ideologías, etiologías, terapéuticas, rituales, hábitos y prácticas únicas) que han sido construidas en ámbitos locales y regionales, y las cuáles pueden tener el riesgo de provocar alteraciones físicas y daños en algunas funciones u órganos del cuerpo humano ♦
